# S11-4 Participatory physical activity promotion in senior residencies

**DOI:** 10.1093/eurpub/ckac093.058

**Published:** 2022-08-29

**Authors:** Dorothee Altmeier

**Affiliations:** Institut für Sportwissenschaft, Universität Tübingen, Tübingen, Germany

**Keywords:** participatory health research, aging, photovoice, senior homes, nursing homes

## Abstract

**Background:**

During their final life decade, people in Germany tend to live in senior residences or nursing homes. Re-location to such institutionalized homes is associated with declining levels of physical activity. Beside intrapersonal factors, like dealing with illness, social and physical environments might be decisive in shaping residents' physical activity levels. At the same time, it remains largely underresearched what residents themselves perceive as barriers and promoters of physical activity. This presentation aims to introduce first results of BaSAlt (BMG, 2019-2022), a participatory research project on physical activity promotion in senior residences in Germany.

**Methods:**

We draw upon data from the BaSAlt study (2019-2022) in eight German senior residences, generated using Photovoice with 48 residents, staff, and visitors, as well as semi-structured interviews (n = 24), group discussions (n = 8), cooperative planning in future workshops (n = 16) and document analysis. Recorded and transcribed interviews, photos, discussions, as well as documents are subject to thematic analysis (Braun & Clarke 2016). All participating actors become part of data collection, and analysis, as well as in developing and implementing change projects to promote physical activity informed by findings.

**Results:**

The presentation will highlight similarities and differences in participants' perceptions of physical activity-related promoters and barriers. We will present a preliminary comparative assessment of the organizational readiness in participating senior residences and potential consequences for cooperative planning in future workshops. Our aim is to initiate concrete action to improve physical activity promotion.

**Conclusions:**

The BaSAlt design recognizes residents, staff, and visitors as experts of their own lives, which allows for greater awareness of people's unique experiences and perceptions. The participatory approach used both for data collection and intervention planning might help uncover physical activity-related promoters and barriers that might otherwise have remained hidden and thus support the development of individualized and targeted action for physical activity promotion.

